# Describing randomisation: patients' and the public's preferences compared with clinicians' practice

**DOI:** 10.1038/sj.bjc.6600527

**Published:** 2002-10-07

**Authors:** V Jenkins, L Leach, L Fallowfield, K Nicholls, A Newsham

**Affiliations:** Cancer Research (UK) Psychosocial Oncology Group, Brighton & Susssex Medical School, University of Sussex, Falmer, Sussex BN1 9QG, UK

**Keywords:** randomisation, cancer, patients' preferences

## Abstract

Explaining the concept of randomisation in simple terms to patients during the discussion of randomised clinical trials can be a difficult task for many health care professionals. We report the results of a questionnaire-based survey, using seven descriptions of randomisation taken from Corbett's study. We examined the preferences of the general public and patients towards the descriptions and compared the results with the clinicians' choice. Participants in the survey were 341 lay people without cancer, 200 patients with cancer and 200 oncologists from cancer centres throughout the UK. It was difficult to identify ‘the best’ way to describe the process of randomisation. The two most favoured statements for patients and members of the public included a very explicit statement that mentioned ‘a computer’, ‘chance’ and ‘not the doctor's or patient's decision’ and a succinct statement that played down the role of ‘chance’. Clinicians chose neither of these statements as closely resembling their own practice. Patients and members of the public most disliked the statement ‘a computer will perform the equivalent of tossing a coin to allocate you to one of two methods of treatment’. This analogy used by 26% of oncologists, was viewed as trivialising and upsetting in the context of determining treatment for life threatening disease.

*British Journal of Cancer* (2002) **87**, 854–858. doi:10.1038/sj.bjc.6600527 www.bjcancer.com

© 2002 Cancer Research UK

## 

There is a large pool of patients eligible to participate in randomised controlled trials (RCT) for new cancer therapies; however, accrual rates are very low (Ward *et al*, 1992). With the recent publication of the House of Commons Report and Proceedings of the Science and Technology Committee entitled ‘Cancer Research – A Fresh Look’ (July 2000), there is increasing pressure to recruit patients into trials. The Committee recommends that: ‘Increasing the number of adult cancer patients entering clinical trials must become a high priority. We recommend that the Government sets challenging and specific targets for the proportion of eligible adult cancer patients entering clinical trials for all the most common cancers’. (Department of Health, 2000)

The Government acknowledges the Committees' recommendation and aims for the newly formed NHS Cancer Research Network to double the number of patients entering trials within 3 years (ibid). It is essential then, to consider and address some of the possible causes for the poor accrual rates. Randomised clinical trials pose particular problems, and the concept of randomisation raises many issues for both health care professionals and patients alike.

For clinicians, the business of discussing randomisation with their patients can be fraught with many difficulties, raising both professional dilemmas and personal conflicts. These include: the pressures created by the changes in the delivery of cancer services (Smyth *et al*, 1994), concern about ethical and medico-legal issues (Shelton, 2001), and the attitudes of both patients (Llewellyn-Thomas *et al*, 1991; Wingerson *et al*, 1993; Fallowfield *et al*, 1998) and doctors (Fallowfield *et al*, 1997; Taylor *et al*, 1984). Some oncologists have highlighted that the actual task of explaining randomisation and obtaining informed consent can be particularly complex in the context of cancer (Fallowfield *et al*, 1998; Jenkins *et al*, 1999).

It is clear that for some patients, understanding the concept of randomisation and its implications is difficult to grasp, leading to confusion and perhaps, reluctance to enter a particular trial (Cook-Gotay, 1991). In a previous study (Corbett *et al*, 1996) favour was found for the less explicit explanations of randomisation, the ones that played down the role of chance. However, these opinions came from a small non-representative group of people and as the authors point out, opinions may differ in patients with a life threatening disease. Doctors acknowledge that they have difficulty in explaining the principles of RCTs to anxious patients seeking reassurance and certainty about optimal treatments. The Science and Technology Committee (Department of Health, 2000) recommends that stronger safeguards are put in place to ensure that informed consent is obtained from all patients before enrolment into a clinical trial. Many have focused on the importance of document readability for patients considering entry to trials (e.g., Hochhauser, 1999; Bjorn *et al*, 1999), while others report that few patients actually read the information sheets (Corbett *et al*, 1996) and that the usefulness of written information correlates highly with the educational level of the patient (Hietanen *et al*, 2000). Previous work by the authors showed that one of the top reasons for patients' accepting trial entry is ‘trust in the doctor’ (Jenkins and Fallowfield, 2000) – implying that the act of communication has a greater influence on the patient's decision than the written word. Therefore, it is vital to try to establish some clear language guidelines to explain randomisation so that a patient can make an educated decision about whether or not to participate in a clinical trial.

This study aims to examine whether there is a preferred way to describe the randomisation process that may facilitate discussions about clinical trials of cancer therapy.

## MATERIALS AND METHODS

### Questionnaire (adapted)

The introduction to each questionnaire began with a short scenario. Members of the public without cancer were asked to: ‘Imagine that you have recently been diagnosed with cancer and have an appointment with a specialist to talk about how to treat your condition. The specialist tells you that there is a research trial going on in the hospital comparing two treatments, both of which are suitable for your illness. However, the only scientific way to compare one treatment with another is for the choice between the two treatments to be made randomly.’ And patients with cancer were asked to: ‘Imagine that you have an appointment with a specialist to talk about how to treat your condition. The specialist tells you there is a research trial going on in the hospital comparing two treatments both of which are suitable for your illness. However, the only scientific way to compare one treatment with another is for the choice between the two treatments to be made randomly.’

Both groups were then presented with seven statements (in a balanced order) explaining randomisation (see Table 1

**Table 1:**
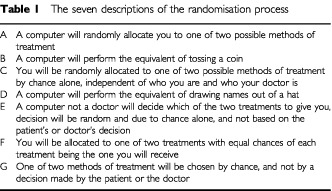
The seven descriptions of the randomisation process

) and were asked to rate the clarity of each statement on a scale of 0–10 (0 being ‘not at all clear’ and 10 being ‘very clear’). Participants were also asked to select their most and least preferred description and give a reason for their choice. Clinicians were sent only the seven statements and asked to select which description best reflected the one they used when discussing trials with patients. A comments box was also provided, allowing the doctors to add their own variations or elaborate on the reasons for their choice, (Corbett *et al*, 1996).

## SAMPLE

### Members of the public

A convenience sample of 341 members of the general public participated in the survey. They were approached by a member of the research team in a variety of public and work places throughout the South East of England, e.g. railway stations, shopping centres etc. Approximately one in three people approached declined to complete the questionnaire.

### Patients

A convenience sample of patients in the local oncology out patient department waiting area was invited to join the study. Two hundred patients participated and 19 patients declined to take part; the reasons for refusal were: ‘no reading glasses with them’ (*n*=6), ‘felt too tired/unwell' (*n*=7), ‘anxious about their appointment’ (*n*=3) and ‘found the questionnaire confusing’ (*n*=3 older patients). The majority of patients were women with breast cancer (23.5%), men with prostate cancer (17.5%) and patients with colorectal cancer (14.5%). The demographic characteristics of both patients and members of the public are shown in Table 2

**Table 2:**
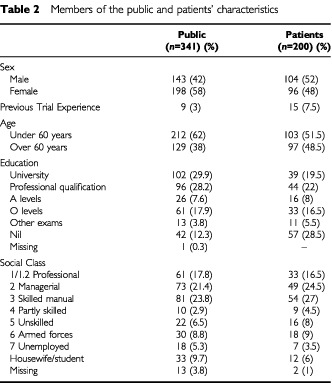
Members of the public and patients' characteristics

.

### Clinicians

Two hundred and sixty-seven clinicians working in UK cancer centres were sent the questionnaire listing the seven statements, and 200 (75%) returned the questionnaire. Table 3

**Table 3:**
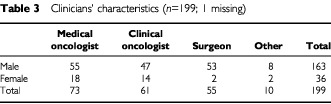
Clinicians' characteristics (*n*=199; 1 missing)

shows the distribution of the group by sex and specialty. The number of trials that clinicians said they were engaged with varied from 0 to 30 (mean five, mode three).

## RESULTS

### Clarity of statements

All participants recorded the clarity for each statement on a scale of 0 to 10. The mean, median, modes are shown in Table 4

**Table 4:**
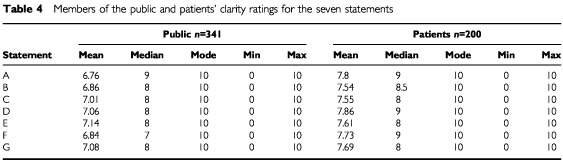
Members of the public and patients' clarity ratings for the seven statements

.

### Members of the public

Unlike Corbett's study (Corbett *et al*, 1996) where a significant advantage was found for Statement ‘F’, members of the public rated all the statements as fairly clear. We examined differences in rating of clarity for each statement by sex and age groups. There were no significant differences in ratings between the men and the women when multiple comparisons were taken into account.

### Patients

Again the data are positively skewed, showing that all the statements appeared clear, but unlike the general public, the patients rated the statements all slightly higher. There were no significant differences between the sexes in the rating of clarity for each statement or differences between age groups.

### Preferred choice of statement

Figure 1

**Figure 1:**
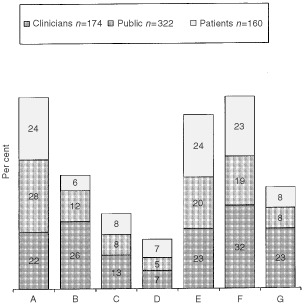
Shows the preferred choice of statements by the clinicians, public and patients.

illustrates the similarities and differences between the choice of statements used by the clinicians and the statements most preferred by the patients and members of the public. Chi square and Student's *t*-tests were applied to the data where appropriate.

### Members of the public

Three hundred and twenty-two members of the public had one preferred statement, nine did not make a choice, six liked them all and four people did not like any of the statements. Of those who did make a choice, 28% (91 out of 322) preferred Statement ‘A’ ‘Once you have agreed to enter the trial, a computer will randomly allocate you to one of two possible methods of treatment.’ This is the most succinct statement with the fewest words. It is the least descriptive as it does not mention ‘chance’, that neither the doctor not the patient will choose the treatment arm, or include an analogy. However, second favourite, chosen by 20% (64 out of 322) of the sample, contained all of these phrases (Statements ‘E’). We examined whether there was a preference for statements according to sex or age group (under or over 60 years). In both age groups Statement ‘A’ was the favourite choice (29% (60 out of 204) and 26% (31 out of 118) respectively) and remained so for both the men (31% (41 out of 132)) and women (26% (50 out of 190)).

The statements least preferred by members of the public were Statement ‘F’ (21% (67 out of 315)) ‘Once you have agreed to enter the trial, you will be allocated to one of two treatments with equal chances of each treatment being the one you will receive’ and Statement ‘B’, ‘Once you have agreed to enter the trial, a computer will perform the equivalent of tossing a coin to allocate you to one of two methods of treatment’ (21% (66 out of 315)). Again we looked at the effect age and sex had on these choices. The younger group (under 60 years) disliked Statement ‘F’ the most (23% (48 out of 204)) but the older age group disliked Statement ‘B’ (24% (27 out of 111)). Women disliked the term ‘tossing a coin’ (22% (40 out of 184)) while the men disliked ‘equal chances’ (23% (30 out of 131)).

### Patients

One hundred and sixty patients chose one statement that they preferred, eight did not make a choice, 14 liked them all and 18 did not like any. Of the patients who made a choice, there were three favourites ‘A’ (24% (38 out of 160)), ‘E’ (24% (39 out of 160)) and ‘F’ (23% (37 out of 160)), similar to the choice of general public. The younger age group had a clear preference for Statement ‘A’ (27% (25 out of 91)) and the older group preferred Statement ‘F’ (29% (20 out of 69)). In addition, men had a preference for Statement ‘A’ (26% (21 out of 82)) and the women preferred Statements ‘E’ (29% (23 out of 78)) and ‘F’ (28% (22 out of 78)). When we looked at sex by age group in a little more detail we noted a difference between the sexes. The younger men nominated Statement ‘A’ as first choice (30% (11 out of 36)) and the younger women chose ‘E’ (31% (17 out of 55)). Similarly there were different preferences in the older age group between the men and women, with men choosing ‘E’ (24% (11 out of 46)) and women overwhelmingly choosing ‘F’ (48% (11 out of 23)).

The patients' least favourite statement was ‘B’ (31% (50 out of 160)), similar to the general public. When we looked at the affect age had on patients' choice of statement we found that both age groups disliked Statement ‘B’ (tossing a coin) (31% (27 out of 88), 32% (23 out of 72)). This statement was equally unpopular with patients of either sex (men 27% (23 out of 84); women 35% (27 out of 76)).

Both the public and patients gave reasons for their choice of statement describing the process of randomisation. The main reason people chose Statement ‘A’ appeared to be because of its brevity and ‘straightforward approach’. Comments included: ‘To the point’, ‘very clear and concise’, ‘short–easy to understand’ and ‘very clear and not patronising’. Whereas those who chose Statement ‘E’ appeared to want more detail, for example ‘it includes all relevant factors – computer, doctor, random, by chance’, ‘it gives more information to me’, ‘it is clear but gives more information as to how the decision is made’, ‘It's slightly longer explanation gives more opportunity for the information to be assimilated'.

When we look at reasons why Statement ‘B’ was overwhelmingly disliked we found that both patients and the public had similar attitudes. One member of the public wrote, ‘If I had cancer I would not like to think of my fate resting on the toss of a coin’, others that it was ‘frightening’, ‘although clear this is trivialising and could be extremely upsetting’. Patients had similar views, ‘too much like a prize draw’, ‘tossing a coin makes it seem like a lottery-which of course it is’. A more suspicious patient wrote, ‘They might use a two headed coin’.

### Clinicians

Two hundred and seventy clinicians were invited to participate in the survey, 200 (74%) responded, (164 men, 36 women). Of these clinicians 129 (64.5%) chose one statement that closely resembled their own practice, 45 chose a combination of statements and 26 provided the phrase they actually used to describe randomisation to patients. Of the 71 clinicians who chose more than one statement or provided their own version, 43 (60%) provided an explanation for their choice. Several stated that they used different phrases according to the patient's (perceived) educational level; others used the term ‘50 : 50 chance’, and some favoured a combination of phrases.

The most frequently chosen description (among the group of 174 clinicians) was Statement ‘F’ ‘Once you have agreed to enter the trial, you will be allocated to one of two treatments with equal chances of each treatment being the one you will receive’. This was chosen by 32% (56 out of 174) of the respondents, closely followed by Statement ‘B’ (26% (45 out of 174)) ‘Once you have agreed to enter the trial, a computer will perform the equivalent of tossing a coin to allocate you to one of two methods of treatment’. When we examined the data by sex and specialty (medical oncologist, clinical oncologist, surgeon and physician) we noted some interesting differences. The most popular choices for women were Statements ‘E’ and ‘F’ (34% (11 out of 32) in each case); whereas men nominated Statements ‘F’ and ‘B’ (32% (45 out of 142) and 28% (40 out of 142) respectively). There were differences in choice between specialties too, with medical oncologists most frequently nominating Statement ‘B’ (27% (18 out of 66)), clinical oncologists nominating Statements ‘F’ and ‘G’ (33% (17 out of 51) in each case) and the surgeons choosing Statement ‘F’ (40% (19 out of 48)). The nominations of the ‘other’ specialty of eight clinicians (chest physicians and palliative care consultants) were more diverse.

Comparison of the clinicians' main choice of statement with that of the public and the patients revealed that Statement ‘F’ was supported by 23% (37 out of 160) of patients and by 19% (60 out of 322) of the public. However, a similar number both of the patients and the public disliked Statement ‘F’ (18% (29 out of 160) and 21% (67 out of 315) respectively), suggesting a polarisation of views. In contrast, clinicians second choice, Statement ‘B’ was nominated as the least favourite description by the majority of patients (31% (50 out of 160) and 21% (66 out of 315)) of the public.

The statement that clinicians voted as the one they most frequently used (F) was voted as unpopular by as many patients and members of the public as those who liked it. The reasons given for its popularity are that ‘no mention of chance or coins – more professional and reassuring – implies equal treatments’, ‘no mention of a computer or randomisation’ and ‘because I feel it will not be a chance decision’. The members of the public who disliked it stated ‘difficult to understand – may confuse a patient’, ‘verbally confusing’, and ‘it does not explain the allocation process and it adds doubt to confusion’. Patients who did not like this explanation, amongst them a journalist and an accountant, said it was ‘gobbledegook’ and ‘waffle’.

## DISCUSSION

Previous research in the area of informed consent has highlighted that the emphasis given to chance in the explanation of the concept of randomisation causes considerable unease amongst patients and the general public (Featherstone and Donovan, 1998; Corbett *et al*, 1996; Fallowfield *et al*, 1998; Jenkins and Fallowfield, 2000). The results from our survey provide clear information on how not to describe the process to patients and potential patients. Specifically, women and older members of the public disliked the common analogy – ‘toss of a coin’, though this was the second most frequently stated phrase amongst our clinicians. Everyone who chose this as their most disliked statement also gave a reason for their choice, which stresses the emotive nature of this phrase. Although using analogies such as flipping coins are expressive and animated, they appear to trivialise the larger issues implicit for patients contemplating cancer trials. Many patients would not anticipate a consultation in which uncertainty and randomisation are discussed, especially at a time when life itself seems so uncertain. Therefore to trivialise the matter, albeit unintentionally, may inadvertently harm the doctor–patient relationship.

In contrast, it was far more difficult to identify ‘the best’ way to describe the process of randomisation. We thought that older people would not like the idea of a ‘computer choosing their treatment’ because as a generation they may be more suspicious of technology. This suggestion was not found to be statistically significant although older patients did favour statements that did not mention a computer more than the younger age group. In addition, there was a polarisation of views for Statement ‘F’ – which clinicians chose as the closest reflection of their own practise. Older patients and women patients preferred it but younger members of the public disliked it.

In contrast to Corbett's study (Corbett *et al*, 1996) there were no significant differences in the ratings of clarity between the statements, suggesting that the wording was simple to read but we did not explicitly check the patients' and public's understanding of the different statements. Also we are aware that participants' read the statements yet during a consultation they would hear them – a very different mode of communication and one that may be more prone to interference. Complementing Corbett's results the statement ‘tossing a coin’ was strongly disliked by both groups of participants. Unfortunately, this term is used very often in hospital patient information sheets to describe the process of randomisation – perhaps it is time to change.

Clinicians and other health professionals are under considerable pressure to provide clear information about clinical trials in an understandable way to patients. This pressure comes not only from Government but also from patient lobby groups. In the Fifth Revision of the Declaration of Helsinki (Wma, (2000)) there were many gains for the rights of consumers. The ‘obligation to ensure people have understood the information provided to them before entering a study’ is one highlighted in an article in the BMJ (Tollman *et al*, 2001). Yet despite all the arguments surrounding trials, there are few practical guidelines available for doctors on how to discuss trials with patients. One group have produced a comprehensive DIY guide on ‘how to do it’ but concluded that the process is not easy and that doctors need to develop good communication skills to enable patients to make a properly informed decision on whether or not to participate in clinical trials (Wager *et al*, 1995).

Unfortunately, a fundamental factor in poor trial recruitment, and the unacceptably low understanding exhibited by patients about the trials they join, is the inadequate communication skills of doctors (Fallowfield and Jenkins, 2001) Discussing trials is extremely difficult and doctors need more education and understanding about patient attitudes as well as training in effective communication skills if the situation is to improve. Flexibility of approach and a better grasp of what members of the public comprehend by words and phrases commonly used by doctors are just one part of the process.

## References

[bib1] BjornERosselPHolmS1999 Can the written information to research subjects be improved? –An empirical study J Med Ethics 25 263267 10390684 10.1136/jme.25.3.263 PMC479221

[bib2] Cook-GotayC1991 Accrual to cancer clinical trials: directions from the research literature Soc Sci Med 33 569577 1962228 10.1016/0277-9536(91)90214-w

[bib3] CorbettFOldhamJLilfordR1996 Offering patients entry in clinical trials: preliminary study of the views of prospective participants J Med Ethics 22 227231 8863148 10.1136/jme.22.4.227 PMC1377002

[bib4] Department of Health2000 Government response to the sixth report of the House of Commons Science and Technology Committee session 1999/2000 Cancer Research – A Fresh Look

[bib5] FallowfieldLJLipkinMHallA1998 Teaching senior oncologists communication skills: results from phase 1 of a comprehensive longitudinal program in the UK J Clin Oncol 16 19611968 9586916 10.1200/JCO.1998.16.5.1961

[bib6] FallowfieldLJRatcliffeDSouhamiRL1997 Clinicians' Attitudes to Clinical Trials of Cancer Therapy Eur J Cancer 33 22212229 9470810 10.1016/s0959-8049(97)00253-0

[bib7] FallowfieldLJenkinsV2001 CommunicationInOxford Textbook of Oncology2nd ednSouhami RL, Tannock I., Hohenberger P, Horiot J-C (eds)pp10491059Oxford: Oxford University Press

[bib8] FeatherstoneKDonovanJL1998 Random allocation or allocation at random? Patients' perspectives of participation in a randomised controlled trial Br Med J 317 11771180 9794849 10.1136/bmj.317.7167.1177 PMC28698

[bib9] HietanenPAroARHolliKAbsetzP2000 Information and communication in the context of a clinical trial Eur J Cancer 36 20962104 11044647 10.1016/s0959-8049(00)00191-x

[bib10] HochhauserM1999 Informed consent and patient's rights documents: a right, a rite, or a rewrite? Ethics Behav 9 120 11657485 10.1207/s15327019eb0901_1

[bib11] JenkinsVFallowfieldL2000 Reasons for accepting or declining to participate in randomized trials for cancer therapy Br J Cancer 821117831788 10839291 10.1054/bjoc.2000.1142 PMC2363224

[bib12] JenkinsVAFallowfieldLJSouhamiASawtellM1999 How do doctors explain RCTs to their patients Eur J Cancer 35 11871193 10615228 10.1016/s0959-8049(99)00116-1

[bib13] Llewellyn-ThomasHAMcGrealMJTheilECFineSErlichmanC1991 Patients' willingness to enter clinical trials: measuring the association with perceived benefit and preference for decision participation Soc Sci Med 32 3542 2008619 10.1016/0277-9536(91)90124-u

[bib14] SheltonJ2001 Letter The Lancet 358 839840

[bib15] SmythJFMossmanJHallRHepburnSPinkertonRRichardsMThatcherNBoxJ1994 Conducting clinical research in the new NHS Br Med J 309 4574617920132

[bib16] TaylorKMMargoleseRGSoskolneCL1984 Physicians' reasons for not entering eligible patients in a randomised clinical trial of surgery for breast cancer N Engl J Med 310 13631367 6717508 10.1056/NEJM198405243102106

[bib17] TollmanSMBastianHDollRHirschLJGuessHA2001 What are the effects of the fifth revision of the Declaration of Helsinki? Br Med J 323 14171423 11744569 10.1136/bmj.323.7326.1417 PMC1121866

[bib18] WagerETooleyPJEmanuelMBWoodSF1995 How to do it. Get patients' consent to enter clinical trials Br Med J 311 734737 7549691 10.1136/bmj.311.7007.734 PMC2550724

[bib19] WardLCFieldingJWDunnJAKellyKA1992 The selection of cases for randomised trials: a registry survey of concurrent trial and non-trial patients. The British Stomach Cancer Group Br J Cancer 66 943950 1419641 10.1038/bjc.1992.390 PMC1977993

[bib20] WingersonDSullivanMDagerSFlickSDunnerDRoy-ByrneP1993 Personality traits and early discontinuation from clinical trials in anxious patients J Clin Psychopharmacol 13 194197 8102621

[bib21] WMA2000 World Medical Association Declaration of Helsinki: ethical principles for medical research involving human subjects JAMA 284 30433045 11122593

